# Variation in *Mycobacterium bovis* genetic richness suggests that inwards cattle movements are a more important source of infection in beef herds than in dairy herds

**DOI:** 10.1186/s12866-019-1530-7

**Published:** 2019-07-05

**Authors:** M. G. Milne, J. Graham, A. Allen, C. McCormick, E. Presho, R. Skuce, A. W. Byrne

**Affiliations:** 1 Veterinary Sciences Division, Agri-food and Biosciences Institute (AFBI), 12 Stoney Road, Stormont, Belfast, BT4 3SD UK; 2 Department of Agriculture, Environment and Rural Affairs (DAERA), Veterinary Service Animal Health, Coleraine, UK; 3 School of Biological Sciences, Queen’s University, Belfast, UK; 4 Present Address: Surveillance, Animal By-Products, and TSEs (SAT) Division, Department of Agriculture, Food and Marine (DAFM), Agriculture House, Dublin 2, Ireland

**Keywords:** Bovine tuberculosis, Northern Ireland, Genotype richness, MLVA, Dairy, Beef

## Abstract

**Background:**

We used genetic Multi-Locus VNTR Analysis (MLVA) data gathered from surveillance efforts to better understand the ongoing bovine tuberculosis (bTB) epidemic in Northern Irish cattle herds. We modelled the factors associated with *Mycobacterium bovis* MLVA genotype richness at three analytical scales; breakdown level, herd level, and patch level, and compared the results between dairy and non-dairy production types.

**Results:**

In 83% of breakdowns and in 63% of herds, a single MLVA genotype was isolated. Five or more MLVA genotypes were found in less than 3 % of herds. Herd size and the total number of reactors were important explanatory variables, suggesting that increasing MLVA genotype richness was positively related to increases in the number of host animals. Despite their smaller relative size, however, the highest MLVA genotype richness values were observed in non-dairy herds. Increasing inwards cattle movements were important positive predictors of MLVA genotype richness, but mainly in non-dairy settings.

**Conclusions:**

The principal finding is that low MLVA genotype richness indicates that small-scale epidemics, e.g. wildlife, contiguous farms, and within-herd recrudescence, are important routes of *M. bovis* infection in cattle herds. We hypothesise that these mechanisms will maintain, but may not explicitly increase, MLVA genotype richness. The presence of elevated MLVA richness is relatively rare and likely indicates beef fattening enterprises, which purchase cattle from over long distances. Cattle movements were furthermore an important predictor of MLVA genotype richness in non-dairy herds, but not in dairy herds; this may represent reduced cattle purchasing levels in dairy enterprises, compared to beef. These observations allude to the relative contribution of different routes of bTB infection between production types; we posit that infection associated with local factors may be more evident in dairy herds than beef herds, however in beef herds, inwards movements offer additional opportunities for introducing *M. bovis* into the herd.

**Electronic supplementary material:**

The online version of this article (10.1186/s12866-019-1530-7) contains supplementary material, which is available to authorized users.

## Background

Genetic approaches such as spoligotyping, MLVA genotyping, and more recently, whole genome sequencing (WGS) are routinely used to help answer epidemiological questions [1–4]. The molecular characterisation of infectious agents can be used to better understand disease outbreak, spread and maintenance, and to better inform interventions [5–7]. In Northern Ireland (NI), MLVA genotyping [1, 8] is a molecular epidemiological tool deployed as a surveillence measure to investigate the ongoing bovine tuberculosis (bTB) epidemic. *Mycobacterium bovis* is the causative agent of bTB, a serious bacterial disease of cattle and livestock. The animal and human health implications of bTB infection are considerable, and far-reaching economic costs are associated with disease control. As yet, however, eradication has remained elusive throughout much of the United Kingdom (UK) and Republic of Ireland (ROI) [9, 10].

MLVA genotyping is carried out on *M. bovis* isolates from culture-confirmed bTB cases, including reactor cattle which test positive to the Single Intradermal Comparative Cervical Tuberculin (SICCT) test, and lesioned animals identified during meat inspection [11]. The information gained from these surveillance activities is used to undertake epidemiological investigations. For example, different *M. bovis* genotypes are associated with relatively different proportions of lesioned reactors in cattle [12] and increasing within-herd *M. bovis* genotype richness (i.e. numbers of different MLVA genotypes) is linked to prolonged and recurrent bTB breakdowns [13].

The *M. bovis* MLVA genotype data also reveals that the genetic structure of the *M. bovis* population in NI is spatially aggregated [14, 15] a phenomenon that has also been observed in *M. bovis* populations in cattle elsewhere [16–19]. Furthermore, *M. bovis* MLVA genotypes are co-localised between cattle hosts and a known wildlife reservoir, the European badger (*Meles meles*) [15]. This spatial clustering suggests that bTB infection may be associated with small scale, local epidemics which maintain (but do not necessarily increase) *M. bovis* MLVA genotype richness. Such transmission routes may include spillback from infected wildlife in the area [4, 20], recrudescence of *M. bovis* MLVA genotypes within-herd [21], infection from a contaminated environment [22], or from contiguous herds [23, 24]. Notwithstanding these processes, increases in *M. bovis* MLVA genotype richness have also been observed within herds [13], alluding to other potential routes of infection. Long-range cattle movements [25, 26], or spreading of contaminated slurry sourced from different areas [27, 28] also present sources of infection, potentially associated with the introduction of additional MLVA genotypes. It is possible that on rare occasions, new *M. bovis* MLVA genotypes may also be introduced via infected wildlife [29]; badgers occasionally undertake long-range movements, which may facilitate wider dissemination of infection [30]. However, the different routes of infection are not mutually exclusive, and their relative contribution to the infection burden is unknown in the context of NI. In GB, however, it is estimated that 16% of herd infections arise from cattle movements, with 75% of infection arising from “local effects”, including badgers and contiguous spread [31].

Different mechanisms of infection may furthermore be associated with different herd management practices. In GB, there is evidence that beef fattening farms may be at more risk of acquiring infection via inwards cattle purchases, whereas dairy farms are more likely to spread infection via the onwards sale of animals [26]. In NI, the purchase of beef cattle is associated with increased risk of bTB breakdown [28], and thus in beef settings, inwards cattle movements may be a relatively more important source of infection than in dairy settings. Furthermore, Lahuerta-Marin et al., [32] found evidence to suggest that in chronically infected herd in NI, the performance characteristics of the skin test and the interferon-gamma test may be poorer in dairy herds relative to beef herds [32]. In GB and NI, dairy reactor animals were less likely to have confirmed infection at *post-mortem* than non-dairy animals [33, 34]. Dairy herds may therefore be at increased risk of persistent and recurrent breakdowns [13, 35, 36], a phenomenon associated with the problem of fully clearing infection using *ante-mortem* diagnostics [37]. Together, this uggests a reduced ability to eradicate infection in dairy settings compared to beef settings and thus, within-herd recrudescence may be a more evident route of infection in dairy herds. As yet, however, the relative contribution of different mechanisms of bTB infection in different herd types in NI is not well understood. What follows, therefore, is the quantification of *M. bovis* MLVA genotype richness across multiple analytical scales in NI, in both dairy and non-dairy settings. We determine the factors associated with this observed richness, and use the results to better understand infection dynamics across both production types.

## Results

### Summary statistics

**Fig. 1:**
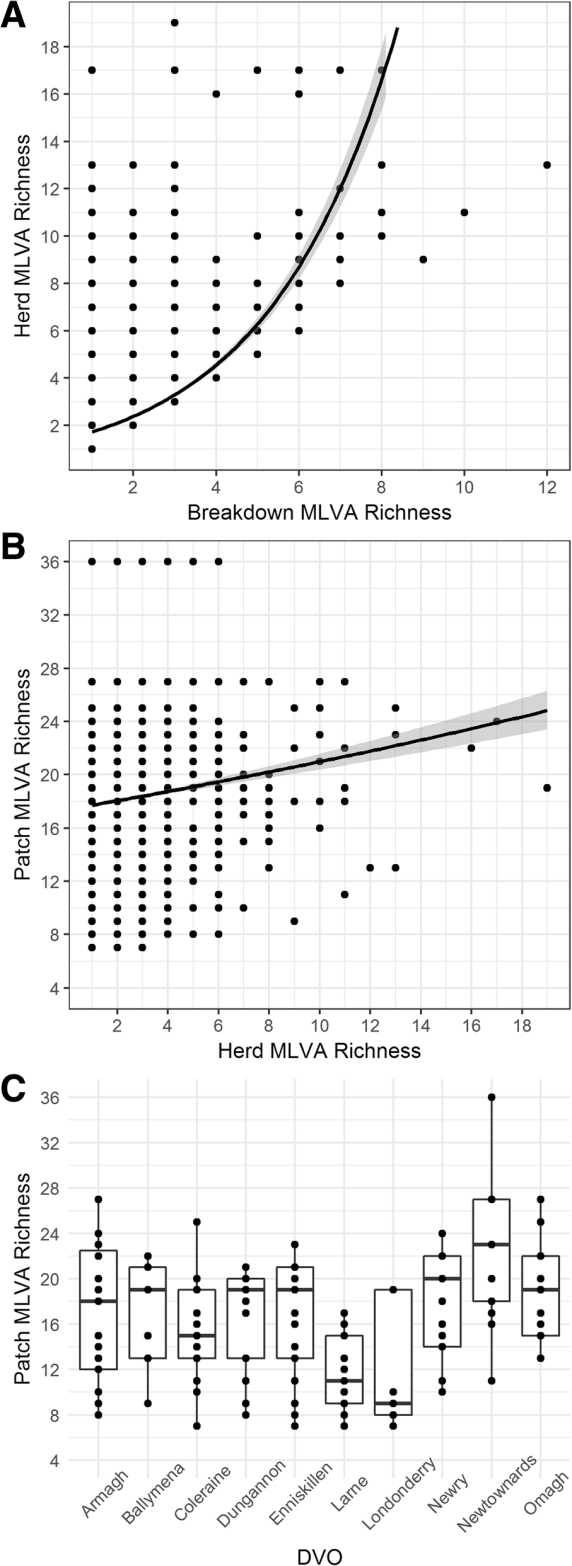
**a-c** The relationship between (**a**) breakdown level MLVA richness and herd level MLVA richness with the fitted Poisson regression line, (**b**) herd level MLVA richness and breakdown level MLVA richness with the fitted Poisson regression line, and (**c**) the distribution of patch level MLVA richness (dots) within each of the ten DVO areas

Final datasets were derived from 29,473 animal-level tests, associated with 7478 bTB breakdowns from 5378 herds. There was moderate correlation between breakdown level MLVA richness and herd level MLVA richness (Spearman’s Rank (R_s_) = 0.52, *p* < 0.05) and every unit increase in breakdown level MLVA richness was associated with an increase in herd level MLVA richness of 38% (IRR:1.38, 95%CI: 1.36–1.40, Fig. 1a). Only weak correlation (R_s_ = 0.08, p < 0.05) was identified between individual herd level richness and patch level MLVA richness, with a 2.1% increase in patch level richness for a unit increases in herd level richness (Univariable Poisson Regression, IRR: 1.02, 95%CI: 1.01–1.03, Fig. 1b). Instead, the mean herd level MLVA richness value exhibited a stronger association with patch level metrics of richness (R_s_ = 0.40, p < 0.05) and a greater effect size (Univariable Poisson Regression, IRR: 1.35, 95%CI: 1.22–1.49). Measures of MLVA genotype richness at the breakdown, herd and patch level varied spatially across NI. Fig. 1c illustrates the broad variation in patch level MLVA genotypic richness in each DVO. Armagh DVO was associated with the highest mean breakdown level and herd level MLVA genotypic richness. However, the highest patch level MLVA genotypic richness was observed in Newtownards DVO. Londonderry DVO was associated with the lowest breakdown level MLVA genotypic richness, herd-level genotypic richness, and patch-level genotypic richness (Table 2).

Exploratory analysis revealed differences in the distribution of explanatory variables between herds with milk licences, and herds without. Whilst the mean herd size at time of breakdown was 141 animals (Standard Deviation (SD) ±146), herds with milk licences (242 animals, SD ± 171) were significantly larger than beef herds (95 animals, SD ± 104; Univariable Poisson Regression, IRR: 2.55, 95%CI: 2.54–2.56). In 40% of breakdowns (*n* = 2964), there were two or fewer reactors disclosed; the majority of these breakdowns (78%, *n* = 2300) occurred in herds without a milk licence. However, the total number of reactors variable did not exhibit the same distribution between dairy and non-dairy herds; increases in the number of reactors disclosed during a breakdown had a positive association with the presence of a milk licence (Univariable Poisson Regression, IRR: 1.7, 95%CI: 1.68–1.73). The presence of a milk licence was also significantly negatively associated with inwards movement intensity (Univariable Poisson Regression, IRR: 0.21, 95%CI: 0.20–0.22).

### MLVA genotype richness at the breakdown level

**Fig. 2:**
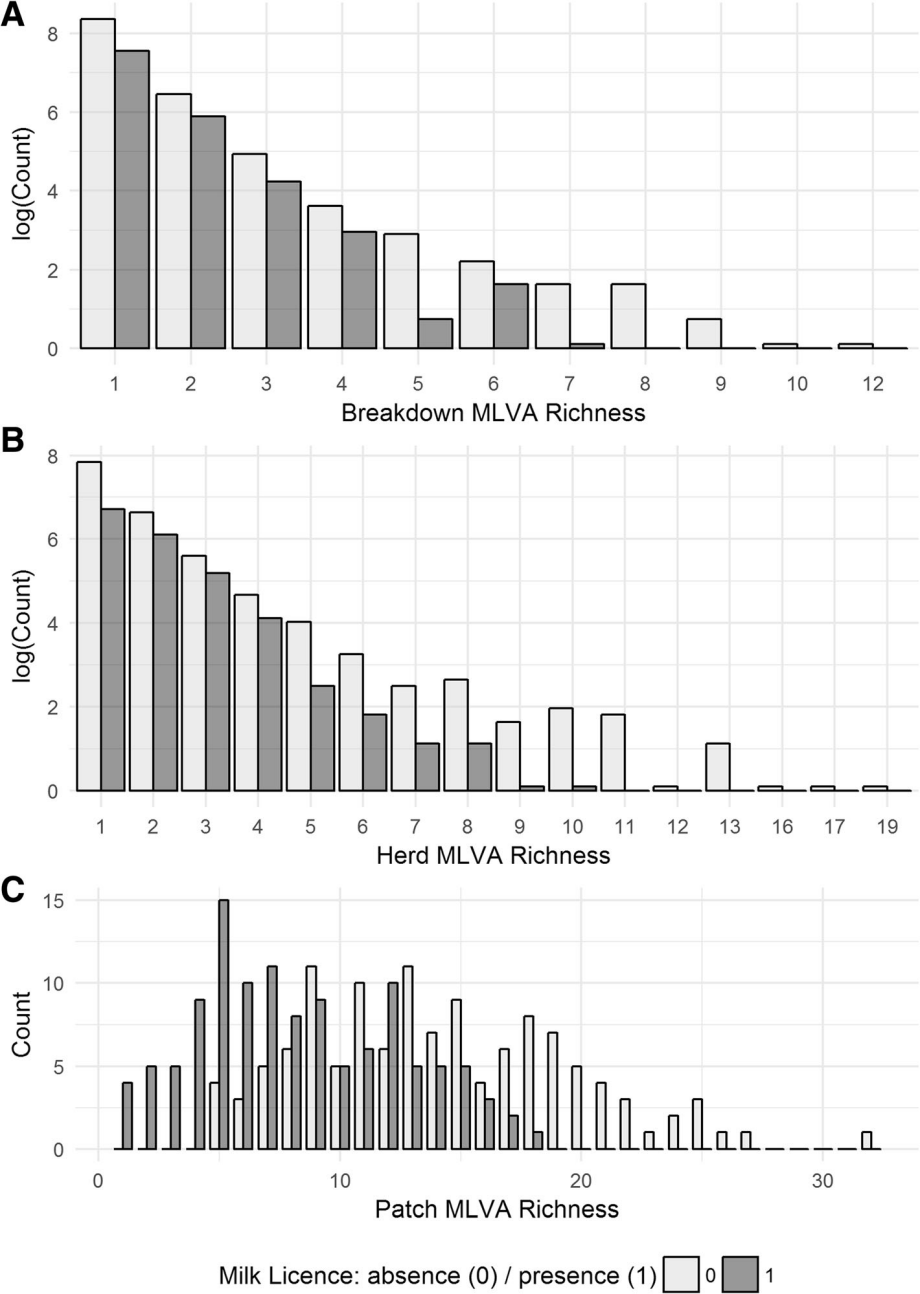
**a-c** The range and frequency of MLVA genotypic richness at three different scales (**a**) breakdown level (**b**) herd level and (**c**) patch level

In 82.5% of all breakdowns (*n* = 6168), only one MLVA type was isolated. The maximum MLVA genotype richness observed was 12 (*n* = 1), and 49 breakdowns (0.66%) yielded 5 or more different MLVA types. Whilst the presence of a milk licence was a positive predictor of MLVA richness, it was not statistically significant (Univariable Poisson Regression: IRR: 1.01, 95%CI: 0.96–1.05). Furthermore, non-dairy herds exhibited greater accumulation of MLVA genotypes. For example, the maximum number of different MLVA genotypes isolated from a breakdown in a non-dairy herd was 12, with 41 breakdowns yielding five or more different MLVA genotypes. In breakdowns in dairy herds however, the maximum number of MLVA genotypes isolated was seven, and only eight breakdowns yielded five or more different MLVA genotypes (Fig. 2a).

All models of breakdown level MLVA genotype richness performed better than “null models” containing the variable for the total number of reactors disclosed during a breakdown (Additional file 1: Table S3). Five main effect explanatory variables were included in the final model for MLVA genotype richness at the breakdown level (Table 3 and Additional file 1: Table S4). The total number of reactors over the breakdown, herd size at the time of breakdown, inwards movement intensity, and breakdown length in days were positively associated with MLVA genotype richness. Latitude was negatively associated with MLVA genotype richness. An interaction term between herd size and inwards movement intensity showed that MLVA genotype richness was elevated in breakdowns in larger herds with large numbers of cattle purchases.

When bTB breakdown data from herds with a milk licence were analysed independently, three explanatory variables were included in the final model (Table 3 and Additional file 1: Table S5); the total number of bTB reactors over the breakdown, herd size at the time of breakdown, and breakdown length in days. The model constructed using data from breakdowns in non-dairy herds (Table 3 and Additional file 1: Table S6) contained four fixed effect explanatory variables and one interaction term. The total number of reactors over the breakdown, herd size at the time of breakdown, inwards movement intensity, and the breakdown length in days were positively associated with MLVA genotype richness. An interaction between herd size and inwards movement intensity showed that larger breakdown herds, with more inwards cattle movements before the bTB breakdown, were furthermore associated with elevated MLVA genotype richness.

### MLVA genotype richness at the herd level

After data were aggregated to herd level, no increases in MLVA genotype richness were observed in 70.6% of herds (*n* = 3795). However only one breakdown occurred in the majority of herds in the dataset (70.9%; *n* = 3803). In those herds with more than one breakdown (*n* = 1575), no increase in MLVA genotype richness was observed in 26.3% of cases (*n* = 414). In 57 herds (3.62%) which experienced more than one breakdown, the number of MLVA genotypes in the breakdown level analysis and the herd level analysis increased by five or more.

Single MLVA isolates were observed in 62.9% of herds (*n* = 3382) and the maximum number of MLVA genotypes isolated was 19 (n = 1), with 159 herds (2.96%) yielding five or more MLVA genotypes. In this analysis, the presence of a milk licence also exhibited a slight association with increasing MLVA genotype richness (Univariable Poisson Regression, IRR: 1.06, 95%CI: 1.02–1.1). Despite this, there was greater accumulation in the overall number of MLVA genotypes in non-dairy herds, as 133 non-dairy herds (3.5%) were associated with between five and 19 different MLVA genotypes, compared to the 26 dairy herds (1.7%) that were associated with between five and 10 MLVA genotypes (Fig. 2b).

The final model for MLVA genotype richness at the herd level for all herds in the dataset contained six main effect variables and two interactions (Table 3 and Additional file 1: Table S7). Thus, the total number of reactors over the breakdown, herd size at the time of breakdown, the count of bTB breakdowns, inwards movement intensity, and the mean breakdown length in days were all positively associated with MLVA genotype richness at the herd level. The number of animal-level *M. bovis* isolates of the most common MLVA genotype found in the herd was instead negatively associated with MLVA genotype richness. A positive interaction between mean herd size and inwards movement intensity indicated that MLVA genotype richness is amplified in larger herds which are also engaged with high levels of inwards cattle purchases. A negative interaction between breakdown length and herd size suggested that larger herds which experienced longer breakdowns were associated with fewer *M. bovis* genotypes than smaller herds which experienced longer breakdowns.

The final model of MLVA genotype richness for herds with a milk licence consisted of six main effect variables and one interaction (Table 3 & Additional file 1: Table S8). Here, the total number of reactors over the breakdown, herd size at the time of breakdown, the count of bTB breakdowns, inwards movement intensity, and the mean breakdown length in days were positively associated with MLVA genotype richness. As with the full model, the number of animal-level *M. bovis* isolates of the most common MLVA genotype was negatively associated with MLVA genotype richness in these herds. Larger herds with higher levels of inwards movement intensity were also associated with elevated herd-level MLVA genotype richness.

In non-dairy herds (Table 3 and Additional file 1: Table S9), MLVA genotype richness was positively associated with the total number of reactors over the breakdown, herd size at the time of breakdown, the count of bTB breakdowns, inwards movement intensity, and the mean breakdown length in days. Two interactions were identified; one interaction showed that larger herds with higher levels of inwards movement intensity were associated with higher herd-level MLVA genotype richness. The second interaction suggested that longer breakdowns in larger herds were associated with less MLVA genotype richness than in smaller herds.

### MLVA genotype richness at the patch level

**Fig. 3:**
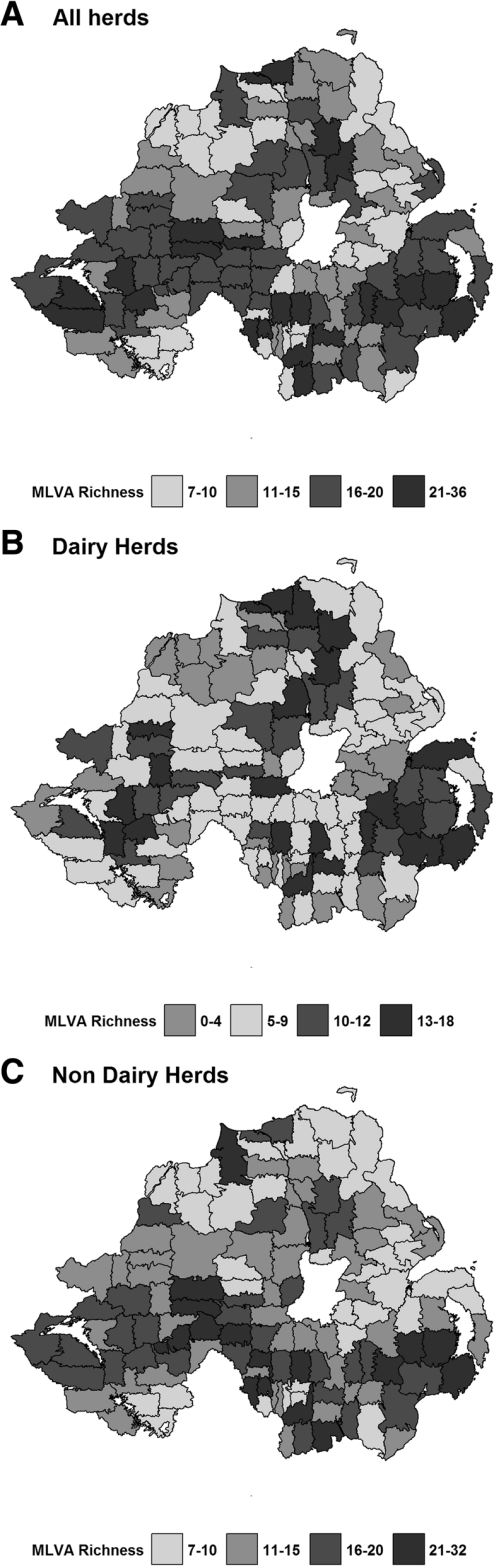
**a-b** MLVA genotype richness metrics at the patch level across Northern Ireland for (**a**) all herds, (**b**) dairy herds and (**c**) non-dairy herds. This material is based upon Crown Copyright and is reproduced and modified with the permission of the Department of Agriculture, Environment and Rural Affairs (DAERA)

There were no patches identified with single isolates, however 23.6% of patches (*n* = 29) were associated with 10 or fewer different MLVA genotype isolates, and 82.1% of patches (*n* = 101) were associated with 20 or fewer different MLVA genotype isolates. Comparing patch level MLVA richness between dairy and non-dairy herds (Fig. 3a-c) revealed a lower median number of isolates when assessing richness in the dairy herds only (median = 8, IQR: 5–12) compared to the non-dairy herds (median = 14, IQR: 10–18). Patch level richness in dairy herds was also found to be significantly lower than patch level richness in non-dairy herds (Univariable Poisson Regression: IRR: 0.58, 95%CI: 0.52–0.64).

The explanatory model for patch-level MLVA genotype richness contained three fixed effect variables (Table 3 & Additional file 1: Table S10); the number of reactors in the patch, total inwards movement intensity in the patch, and mean herd level MLVA genotype richness. When patch-level MLVA genotype richness was derived using only data from those herds with milk licences, the final model consisted of three explanatory variables (Table 3 & Additional file 1: Table S11); the number of reactors in the patch, the total number of cattle on the patch, and the mean herd-level MLVA genotype richness value of the patch. Metrics of patch-level MLVA genotype richness from herds without milk-licences were also associated with three variables (Table 3 and Additional file 1: Table S12); the number of reactors in the patch, total patch-level inwards movement intensity, and the mean herd-level MLVA genotype richness value of the patch.

## Discussion

The results reveal that a single MLVA genotype was isolated in the majority of cases (i.e. 83% of breakdowns, and 63% of herds). The accumulation of MLVA genotype richness at the breakdown level and at the herd level was less common; less than 1 % of breakdowns and 3 % of herds were associated with five or more MLVA genotypes, respectively. Our principal finding is, therefore, that *M. bovis* infection is largely related to a single source of infection, or to multiple local sources of infection associated with the same MLVA genotype. These may include nearby infected wildlife, infected cattle on neighbouring contiguous farms [46], contamination from the environment, or recrudescence of infection associated with MLVA genotypes circulating within the herd [13, 21, 35, 36, 47]. In GB, the majority of *M. bovis* infection was also related to “local” factors such as wildlife and contiguous spread [31]; our results would appear to be in agreement with these findings. We also found that the number of breakdowns was positively associated with MLVA genotype richness, indicating that in some cases, *M. bovis* infection does appear to be associated with the introduction of new MLVA genotypes. However, the vast majority of herds in our study (71%) experienced only one breakdown during the study period. It would therefore appear that the occurrence of breakdowns associated with newly introduced MLVA genotypes is less common. Furthermore, in 26% of herds which did experience more than one breakdown, there were no increases in MLVA genotype richness over successive restriction periods. These results further highlight the relative importance of small-scale, local effects in maintaining the epidemic.

The *M. bovis* population in NI is spatially clustered, with MVLA genotypes localised to geographic areas [14]. Consequently, the accumulation of MLVA genotype richness in herds is indicative of purchasing cattle from a wide geographic extent [26], or possibly the spreading of non-local contaminated slurry [28]. However, elevated MLVA richness was observed in only a limited number of cases, suggesting that a process maintains richness in some herds without the wider dissemination of *M. bovis* genotypes. We posit that elevated MLVA genotype richness within-herd implies that these herds are beef fattening enterprises. These herds operate via the purchase of a large number of cattle, which are then raised for sale, straight to slaughter. This affords the opportunity for the accumulation of MLVA genotype richness with less risk of spreading infection to other herds via the onwards sale of cattle. In GB, beef fattening herds are indeed shown to have a higher turnover of animals than dairy herds [26] which may facilitate the introduction of novel MLVA genotypes. In beef fattening enterprises, the purchase of infected cattle therefore appears to serve as a particularly important source of *M. bovis* infection, in addition to wildlife, nearby farms, within herd recrudescence and environmental contamination.

The low MLVA genotype richness observed in most herds does not mean that cattle movements do not contribute more widely to the epidemic; cattle movements are a known risk factor for bTB in NI [48]. Indeed, a main finding from this work is that higher levels of inwards movement intensity were associated with increasing MLVA genotype richness in non-dairy herds. The impact of inwards cattle movements on MLVA genotype richness in dairy herds was less evident. Previous work from GB revealed differences between purchasing patterns in beef and dairy settings, with dairy herds receiving fewer inwards movements than beef herds [26]; our findings would therefore appear to be in accord with these results. We argue that cattle movements may increase the risk of bTB breakdown, but may not always be important for introducing new MLVA genotypes in dairy herds. Thus, if most cattle purchases consist of short range movements (as opposed to buying and selling cattle from further afield), the same MLVA genotypes will circulate between herds, therefore maintaining current herd level MLVA genotype richness (but not increasing it). We further hypothesise that the spatially structured nature of the *M. bovis* population [14] means that disentangling the relative contributions of local infection routes is exceptionally challenging; a higher resolution phylodynamics approach such as whole genome sequencing may shed more light on this issue [4].

Whilst there was a moderate correlation between breakdown level and patch level metrics of MLVA genotype richness, individual herd level MLVA richness values exhibited only weak correlation with the overall patch level MLVA richness. Higher patch level MLVA richness will indeed partially reflect the larger host population size in patches compared to herds, but it also further shows that herds within a patch share some, but not all, of the same MLVA genotypes. This further hints at the importance of localised infection mechanisms in maintaining MLVA richness within herds. In addition, we found that the mean herd level MLVA richness in the patch did indeed exhibit a stronger association with patch level MLVA richness. This is likely due to the influence of herds with elevated MLVA type richness (i.e. beef fattening herds) impacting on the mean.

In all models, the total number of reactors disclosed during a breakdown was an important explanatory variable for MLVA genotype richness. Thus, at least in part, the accumulation of different *M. bovis* MLVA genotypes is likely to be related to increases in the number of hosts. Increases in herd size were also positively associated with MLVA genotype richness; this may be a further manifestation of the positive relationship between MLVA genotype richness and increasing cattle numbers. However, large herds may also be linked to other factors which afford more opportunities for the accumulation of new *M. bovis* MLVA genotypes e.g. inwards cattle movements, or a larger geographic footprint.

The results furthermore demonstrate that dairy herds were both larger, and contained more reactors, than herds without a milk licence. There was also evidence that at the herd level, the presence of a milk licence had a slight positive association with increased MVLA genotype richness. In spite of this, the highest MLVA genotype richness values were not found in dairy settings. Previous studies show that dairy herds in NI are associated with elevated risk of bTB breakdown compared to other herd types [49] and the diagnostic SICCT can perform relatively poorly in NI dairy settings [32]. Together, this suggests a failure to clear infected animals within dairy herds, thus increasing the likelihood of within herd recrudesce of the same MLVA genotypes. Other factors associated with dairy herds may also limit the opportunity for MLVA genotype accumulation, for example, if dairy farms are less expansive, less fragmented, utilise less shared grazing, or share fewer boundaries with neighbouring farms. As yet however, how farm fragmentation factors and grazing practices influence bTB risk across different herd types is presently unknown in the NI context.

The herd-level analysis suggested that increasing numbers of the most common MLVA genotype found in dairy herds was negatively associated with MLVA genotype richness at the herd level. We consider that rapid propagation of a single MLVA genotype within-herd could arise if within-herd transmission dynamics were different between beef and dairy herds [50]. In dairy settings, factors such as differences in stress profiles [51], higher stocking density, or increased access to shared housing or feed [24, 52], may provide opportunity for the rapid amplification of within-herd bTB infection. However, rates of within-herd transmission, and the factors influencing this metric in cattle herds in NI are not yet known.

## Conclusion

This work alludes to the fact that different routes of infection may be of greater relative importance to different production types in NI. In the majority of herds, there was relatively low MLVA genotype richness, indicating that highly localised factors operating over a small geographical extent were primarily influencing the epidemic, e.g. infection from neighbouring herds, wildlife or within herd recrudescence. Only a limited number of herds were identified with elevated MLVA richness, and these were likely beef fattening enterprises, wherein long range cattle purchases may also represent an important source of new MLVA genotypes. Indeed, inwards movement intensity was an important predictor of increasing MLVA genotype richness in non-dairy settings. Cattle movements appear to be relatively less important for introducing MLVA genotypes into dairy herds, which may be a consequence of less movement intensity associated with dairy herds more generally, and/or the purchase of cattle from the local area which are more likely to share the same MLVA genotypes. Despite the larger number of potential *M. bovis* hosts, the maximum MLVA genotype accumulation was not observed in dairy herds. We therefore posit that in dairy herds, localised infection routes which do not explicitly introduce MLVA genotypes from outside the area, e.g. within herd recrudescence, are more evident. Non dairy herds, however, may also be exposed to additional infection via cattle purchasing. Future eradication should focus on understanding the contribution of infection pathways in different production types. An enhanced understanding of the NI cattle movement network will provide a better understanding of the risk associated with cattle purchasing in different settings. In addition, we advocate that future work should focus on the infection risk associated with farm fragmentation, contiguous herds and shared grazing between different herd types in the Northern Irish context.

## Methods

This retrospective analysis used data collected between 2009 and 2016 inclusive, and was conducted at three analytical scales; breakdown level, herd level and patch level. Cattle herds in NI reside within one of 123 administrative patches, which are situated within 10 Divisional Veterinary Offices (DVOs; Additional file 1: Figure S1)**.** The area of NI is approximately 14,000 km^2^ and mean patch size is approximately 110 km^2^ (SD ± 53). Data on confirmed breakdowns which occurred between January 2003 to April 2016 were made available from the Animal and Public Health Information System (APHIS) database [38]. BTB breakdowns with missing or erroneous data (e.g. location co-ordinates, start and end dates) were removed. Breakdowns were only included if the start and end date (defined by the date of disclosing test, and the date of at which Officially Tuberculosis Free status was restored) was inclusive of the study period. These data were then associated with animal-level *M. bovis* genotype surveillance data (MLVA) from skin-test reactors and lesioned non-reactor animals identified at slaughter [14]. Briefly, all culture-confirmed bTB cases were further sub-cultured with single colonies, then heat killed to create bacterial cell lysates, which were then used directly as Polymerase Chain Reaction (PCR) templates for all molecular characterisation of pathogen variation. Spoligotyping and genotyping of variable number of tandem repeat (VNTR) loci were undertaken using established methods [14, 39]. Standardised international nomenclature defined at https://www.mbovis.org/ was applied for all spoligotypes in the dataset. Eight VNTR loci across the *M. bovis* genome were genotyped [40]; MV2163B/QUB11B, MV4052/QUB26A, MV2461/ETRB, MV1955/Mtub21, MV1895/QUB1895, MV2165/ETRA, MV2163/QUB11A and MV3232/QUB3232. Prior to 2009, MLVA and spoligotyping were usually only carried out on the first confirmed reactor or lesioned case, whereas post-2009, molecular typing was carried out at animal-level, on all culture-confirmed cases.

### Variable construction

The outcome variable in each analysis was MLVA genotype richness, defined as the number of different MLVA genotypes present at each of the three analytical scales. In this study, reactor cattle were treated as individuals, and different *M. bovis* MLVA genotypes were treated as species. At all three scales, three different models were constructed to reflect different enterprise types. Firstly, data on all herds were analysed together. Next, data from herds with a milk-licence (i.e. dairy units) were analysed separately from herds without a milk-licence (non-dairy herds; e.g. beef herds). Nine models were therefore constructed in total. Seven variables were considered for inclusion in the breakdown level analysis (Table 1). These variables were; herd size at the time of breakdown, bTB breakdown length in days, the total number of SICCT reactor animals identified during the breakdown, and herd latitude and longitude in the form of the Irish six figure grid reference. Inwards movement intensity was defined as the number of animals moved into the herd in the six months prior to breakdown, as a proportion of herd size. The number of isolates from animals with the most common MLVA genotype from the breakdown was also derived (i.e. counts of the most common MLVA genotype). For the herd level analysis, data on bTB breakdowns were aggregated to the herd unit, resulting in ten herd level variables (Additional file 1: Table S1). In brief, the outcome variable was calculated as the total number of different MLVA genotypes present in the herd, throughout all breakdowns. The total number of reactors over the breakdown was summed to give the total number of reactors in the herd. The total number of breakdowns per-herd, and the number of isolates from reactors associated with the most common MLVA genotype were also derived. The mean values for herd size, inwards movement intensity, and breakdown length were also quantified. The maximum inward movement intensity, and breakdown length, were also calculated and included as potential explanatory variables. The herd latitude and longitude were as before. For the patch level analysis, breakdown level data were aggregated to the patch unit; 15 variables were included in this analysis (Additional file 1: Table S2). The patch-level MLVA richness variable was derived from the number of unique MLVA genotypes present in the patch. The total number of herds, the total number of herds with a milk licence, the total number of cattle in breakdown herds, the total number of reactors, the total number of breakdowns, the total inwards movement intensity (total number of inwards movements into the patch, as a proportion of cattle in breakdown herds), and the total number of breakdown days per-patch were calculated. The total number of isolates from reactors associated with the most common MLVA genotype in the patch was also quantified. The mean number of breakdowns per-herd, the mean herd-size, the mean inwards movement intensity, and the mean breakdown length per-herd were also included. The latitude and longitude of each patch centroid was calculated. All analyses were carried out in R [41] and Excel.

**Table 1:** Summary statistics for breakdown level variables

		All breakdowns (*n* = 7478)			Dairy only (*n* = 2361)			Non-Dairy Only (*n* = 5117)		
Variable	Definition	Min	Mean (SD)	Max	Min	Mean (SD)	Max	Min	Mean (SD)	Max
count_MLVA_breakdown (outcome)	MLVA genotype richness	1	1.25 (0.67)	12	1	1.25 (0.6)	7	1	1.24 (0.7)	12
n_common_MLVA_type_breakdown	N. reactors with the most common MLVA	1	3 (5.6)	138	1	4 (7.6)	138	1	2 (4.2)	75
herd_size	herd size at time of breakdown	1	141 (146)	1383	2	242 (171)	1383	1	95 (104)	1156
inwards_movement_intensity	inwards movement intensity 6 months before breakdown	0	0.14 (0.2)	1	0	0.04 (0.08)	0.80	0	0.19 (0.22)	1
breakdown_length_days	bTB breakdown length in days	31	225 (140)	2288	33	230 (141)	1841	31	224 (140)	2288
total_reactors_over_breakdown	total number of reactors during the breakdown	1	8 (13.8)	280	1	11 (18.8)	280	1	6 (10.4)	209
y	Latitude (/100)	3114		4516	3124		4441	3114		4516
x	Longitude (/100)	1898		3646	2105		3635	1898		3646

**Table 2:** Summary of MLVA genotype richness values at the breakdown level, herd level and patch level, on a per-DVO basis

DVO	N. bTB breakdowns	N. herds	N. patches	Mean breakdown-level MLVA richness (SD)	Mean herd-level MLVA richness (SD)	Mean patch-level MLVA richness (SD)
Armagh	639	502	17	1.33 (0.89)	1.82 (1.73)	15.43 (6.06)
Ballymena	444	322	6	1.16 (0.52)	1.51 (0.93)	16.50(5.05)
Coleraine	898	647	12	1.18 (0.67)	1.55 (1.09)	15.00 (6.86)
Dungannon	623	496	14	1.29 (0.73)	1.82 (1.61)	16.00 (4.57)
Enniskillen	970	698	14	1.22 (0.54)	1.50 (0.94)	14.58 (5.77)
Larne	364	254	14	1.19 (0.57)	1.61 (1.18)	11.15 (3.31)
Londonderry/Strabane	209	158	6	1.09 (0.33)	1.39 (0.89)	10.60 (4.83)
Newry	1222	876	16	1.28 (0.70)	1.67 (1.21)	16.81 (5.01)
Newtownards	1016	649	10	1.27 (0.63)	1.76 (1.19)	20.20 (7.02)
Omagh	1093	776	14	1.27 (0.70)	1.72 (1.28)	18.79 (4.00)
*Grand Total/Avg*	7478	5378	123	1.25 (0.67)	1.66 (1.25)	15.75 (5.56)

**Table 3:** Summary of the Incidence Rate Ratio (IRR) estimates and 95% lower (L) and upper (U) confidence intervals for MLVA genotype richness across three scales and two production types

	All data			Dairy only			Non-dairy only		
breakdown-level results									
Variable	Coeff.	95%CI L	95%CI U	Coeff.	95%CI L	95%CI U	Coeff.	95%CI L	95%CI U
total_reactors_over_breakdown	1.07	1.06	1.09	1.10	1.07	1.13	1.07	1.05	1.09
n_common_MLVA_type_breakdown	–	–	–	–	–	–	–	–	–
herd_size	1.08	1.06	1.10	1.05	1.01	1.09	1.06	1.04	1.09
inwards_movement_intensity	1.10	1.07	1.12	–	–	–	1.09	1.06	1.11
breakdown_length_days	1.10	1.09	1.12	1.08	1.05	1.12	1.10	1.08	1.12
y_latitude	0.98	0.96	0.99	–	–	–	–	–	–
herd_size:inwards movement intensity	1.06	1.04	1.08	–	–	–	1.05	1.03	1.07
herd-level results									
sum_reactors_herd	1.10	1.07	1.13	1.13	1.06	1.21	1.08	1.06	1.10
sum_reactors_most_common_MLVA	0.96	0.93	0.99	0.94	0.88	0.99	–	–	–
mean_herd_size	1.17	1.14	1.19	1.13	1.09	1.17	1.13	1.11	1.16
sum_number_breakdowns	1.19	1.17	1.21	1.18	1.14	1.22	1.19	1.16	1.21
mean_inwards_movement_intensity	1.19	1.17	1.21	1.07	1.03	1.11	1.18	1.15	1.21
mean_breakdown_length	1.10	1.08	1.12	1.06	1.02	1.10	1.08	1.06	1.11
mean_herd_size:inwards_movement_intensity	1.10	1.08	1.12	1.04	1.01	1.08	1.07	1.05	1.09
mean_herd_size:mean_breakdown_length	0.98	0.97	0.99	–	–	–	0.98	0.97	0.99
patch-level results									
sum_total_reactors_in_patch	1.23	1.18	1.28	1.11	1.02	1.20	1.24	1.14	1.34
inwards_movement_intensity_patch	1.08	1.03	1.13	–	–	–	1.08	1.00	1.17
count_breakdown_cattle_patch	–	–	–	1.24	1.15	1.35	–	–	–
mean_herd_MLVA_richness	1.07	1.02	1.13	1.14	1.07	1.22	1.14	1.07	1.22

### Modelling

Models were constructed using the *lme4* package [42]. The outcome (counts of MLVA genotypes) was modelled using Poisson regression with a log link. The same modelling procedure was applied to each of the nine models. Firstly, univariable relationships between the outcome and each of the predictor variables were explored. Continuous predictor variables were assessed for correlation as a potential source of multi-colinearity. Variables exhibiting a correlation coefficient > 0.5 or < − 0.5 were considered for removal, whilst retaining predictors with the strongest association with the outcome, as determined by Akaike Information Criterion (AIC) values. The remaining covariates (including biologically plausible interactions) were considered for inclusion in final models. As these data were hierarchical, the most appropriate random effects structure in each model was firstly derived. For the breakdown level analysis, random effects included the herd, the patch and the DVO. For the herd level analysis, potential random effects included the patch and DVO. For the patch level analysis, DVO was the only random effect considered.

Following the procedures outlined in Zurr et al. (2009) [43], initial models were constructed with all potential covariates included in the fixed effects component, along with the appropriate random effects for the given model. Models were also constructed which included fixed effects only. The decision to use random effects was informed by comparing AIC values between models with and without random effects, using both AIC values and by assessing the variance associated with each random effect. Once the random effects structure was determined, AIC values were then used as a guide for model selection following a backwards stepwise process, with smaller AIC values indicating better fitting, more parsimonious models. During model construction, variables were also assessed for confounding effects. Finally, any variables omitted from the modelling process were reintroduced and the impact on AIC values was determined, in order to ensure that all important predictors were included. Models were checked to ensure that over-dispersion was not present. After model construction, models were validating by plotting covariates against residuals to assess for patterns. Residuals were also plotted against covariates which were not included in the model. Finally, models were re-run, excluding data associated with large residual values, and the impact on covariates was assessed. Spatial autocorrelation in the residuals was checked using the *GeoR* package [44] and Moran’s I [45], however no significant evidence of spatial autocorrelation was detected. All predictor variables were scaled and centred at the mean prior to analysis, to permit comparisons of the relative effects of covariates. The exponentiated parameters of the resulting models can be interpreted as Incidence Rate Ratios (IRR), which are reported along with the 95% upper and lower confidence intervals (CI). Final models were also compared to “null” models which contained only the number of reactors variable as the sole predictor (Additional file 1: Table S3).

## Additional file

Additional file 1 **Figure S1.** Patches and Divisional Veterinary Offices (DVOs) within NI. **Table S1.** Summary statistics for herd level variables. **Table S2.** Summary statistics for patch level variables. **Table S3.** AIC values for each of the models, compared to null models containing only the “total reactors” variable. **Table S4.** Final full model for the breakdown level analysis constructed using all data. **Table S5.** Final full model for the breakdown level analysis constructed using only data from herds with milk licences. **Table S6.** Final full model for the breakdown level analysis constructed using only data from herds without milk licences. **Table S7.** Final full model for the herd level analysis constructed using all data. **Table S8.** Final full model for the herd level analysis constructed using data from herds with milk licences. **Table S9.** Final full model for the herd level analysis constructed using data from herds without milk licences. **Table S10.** Final full model for the patch level analysis constructed using data from all herds. **Table S11.** Final full model for the patch level analysis constructed using only data from herds with milk licences. **Table S12.** Final full model for the patch level analysis constructed using only data from herds without milk licences. (DOCX 187 kb)

## Data Availability

The datasets generated and analysed during the current study are not publicly available by request of the data controller (the project funder) who deem these data regarding disease status as sensitive. Any data related enquires should be made to the Department of Agriculture, Environment and Rural Affairs (DAERA; https://www.daera-ni.gov.uk).

## Abbreviations

AIC: Akaike Information Criterion
APHIS: Animal and Public Health Information System
bTB: Bovine tuberculosis
CI: Confidence Interval
DVO: Divisional Veterinary Offices
IRR: Incidence Rate Ratio
MLVA: Multi-Locus VNTR Analysis
NI: Northern Ireland
PCR: Polymerase Chain Reaction
ROI: Republic of Ireland
R_s_: Spearman’s Rank Correlation Coefficient
SD: Standard Deviation
SICCT test: Single Intradermal Comparative Cervical Tuberculin test
UK: United Kingdom
VNTR: Variable Number of Tandem Repeat
WGS: Whole Genome Sequencing
