# Supplementary material for: Variation in Mycobacterium bovis genetic richness suggests that inwards cattle movements are a more important source of infection in beef herds than in dairy herds
Source: BMC Microbiol. 2019 Jul 5;19:154. doi: 10.1186/s12866-019-1530-7 (PMC6612228; doi:10.1186/s12866-019-1530-7)
Supplement: Supplementary file 1 — Figure S1. Patches and Divisional Veterinary Offices (DVOs) within NI. Table S1. Summary statistics for herd level variables. Table S2. Summary statistics for patch level variables. Table S3. AIC values for each of the models, compared to null models containing only the “total reactors” variable. Table S4. Final full model for the breakdown level analysis constructed using all data. Table S5. Final full model for the breakdown level analysis constructed using only data from herds with milk licences. Table S6. Final full model for the breakdown level analysis constructed using only data from herds without milk licences. Table S7. Final full model for the herd level analysis constructed using all data. Table S8. Final full model for the herd level analysis constructed using data from herds with milk licences. Table S9. Final full model for the herd level analysis constructed using data from herds without milk licences. Table S10. Final full model for the patch level analysis constructed using data from all herds. Table S11. Final full model for the patch level analysis constructed using only data from herds with milk licences. Table S12. Final full model for the patch level analysis constructed using only data from herds without milk licences. (DOCX 187 kb) [file 12866_2019_1530_MOESM1_ESM.docx]

**Additional file**

**Variation in *Mycobacterium bovis* genetic richness suggests that inwards cattle movements are a more important source of infection in beef herds than in dairy herds**

**Milne, G.M.^1*^, Graham, J. ^1^, Allen, A. ^1^, McCormick, C. ^1^, Presho, E. ^1^, Skuce, R. ^1^, Byrne, A.W.^1, 2^**

**^1^** Veterinary Sciences Division, Agri-food and Biosciences Institute (AFBI),

12 Stoney Road, Stormont, Belfast BT4 3SD, UK­­­­­

^2^ School of Biological Sciences, Queen’s University Belfast, Belfast, UK.

*corresponding author [georgina.milne@afbini.gov.uk](mailto:georgina.milne@afbini.gov.uk), ORCID https://orcid.org/0000-0002-9023-8500

**
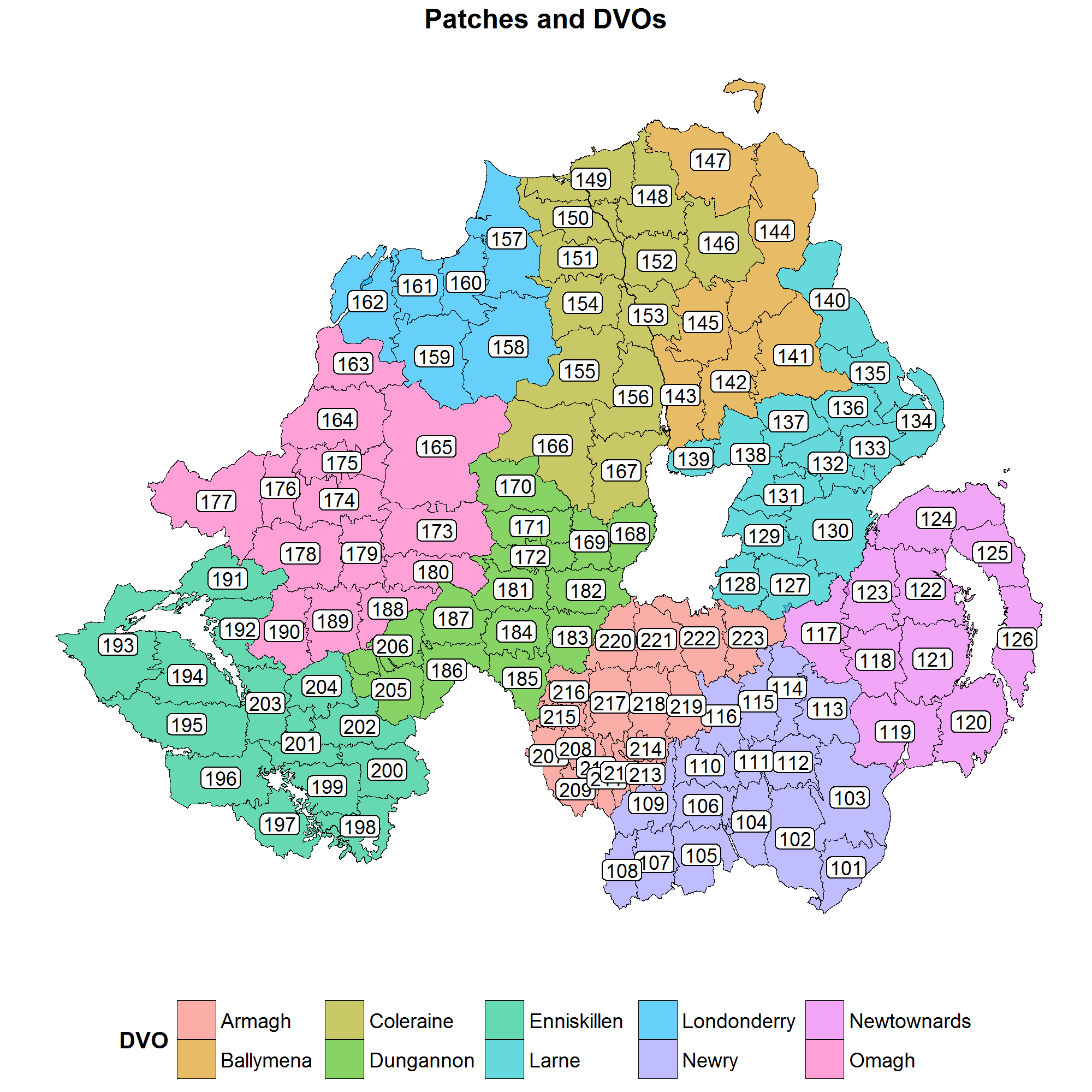
**

**Figure S1:** Patches and Divisional Veterinary Offices (DVOs) within NI. This material is based upon Crown Copyright and is reproduced and modified with the permission of the Department of Agriculture, Environment and Rural Affairs (DAERA).

**Table S1:** Summary statistics for herd level variables.

| **Variable** | **Definition** | **Min** | **Mean (SD)** | **Max** | **Min** | **Mean (SD)** | **Max** | **Min** | **Mean (SD)** | **Max** |
| --- | --- | --- | --- | --- | --- | --- | --- | --- | --- | --- |
|  |  | All herds (n = 5,378) | | | Dairy only (n = 1,543) | | | Non-Dairy Only (n =3,835) | | |
| count_MLVA_herd (outcome) | MLVA genotype richness | 1 | 1.66 (1.25) | 19 | 1 | 1.73 (1.03) | 10 | 1 | 1.63 (1.33) | 19 |
| total_reactors_herd | total number of reactors in the herd | 1 | 11 (19.0) | 300 | 1 | 16 (26.5) | 285 | 1 | 8 (14.2) | 300 |
| count_breakdown_herd | N. bTB breakdowns per-herd | 1 | 1.4 (0.75) | 6 | 1 | 1.5(08) | 6 | 1 | 1.3 (0.7) | 6 |
| n_common_MLVA_type_herd | N. reactors with the most common MLVA type | 1 | 4 (7.1) | 140 | 1 | 5 (10.3) | 140 | 1 | 3 (5.17) | 75 |
| mean_herd_size | mean number of cattle in the herd | 1 | 123 (134) | 1184 | 2 | 226 (167) | 1184 | 1 | 82 (90) | 971 |
| mean_inwards_movement | mean inwards movement intensity 6 months before breakdown | 0 | 0.14 (0.2) | 0.99 | 0 | 0.04 (0.08) | 0.8 | 0 | 0.17 (0.2) | 0.99 |
| max_inwards_movement | max inwards movement intensity 6 months before breakdown | 0 | 0.15 (0.2) | 99.58 | 0 | 0.05 (0.09) | 0.8 | 0 | 0.19 (0.2) | 0.99 |
| mean_breakdown_length | mean bTB breakdown length (days) | 31 | 224 (133) | 2288 | 81 | 230 (131) | 1750 | 64 | 220 (130) | 2288 |
| max_breakdown_length | max bTB breakdown length (days) | 31 | 241 (156) | 2288 | 81 | 257 (160) | 1841 | 64 | 237 (154) | 2288 |
| y | Latitude (/100) | 3114 |  | 4516 | 3124 |  | 4441 | 3114 |  | 4516 |
| x | Longtidude (/100) | 1898 |  | 3646 | 2105 |  | 3635 | 1898 |  | 3646 |

**Table S2:** Summary statistics for patch level variables.

| **Variable** | **Definition** | **Min** | **Mean (SD)** | **Max** | **Min** | **Mean (SD)** | **Max** | **Min** | **Mean (SD)** | **Max** |
| --- | --- | --- | --- | --- | --- | --- | --- | --- | --- | --- |
| count_MLVA_patch (outcome) | MLVA genotype richness | 6 | 15.6 (5.6) | 36 | 0 | 8.2 (4.2) | 18 | 5 | 14.2 (5.5) | 32 |
| mean_count_MLVA_herd | mean herd-level MLVA richness | 1 | 1.66 (0.3) | 3 | 0 | 1.68 (0.4) | 3 | 1 | 1.67 (0.4) | 4 |
| count_herds_patch | total number of herds per-patch | 9 | 44 (23) | 124 | 0 | 13 (9) | 43 | 6 | 31 (20) | 113 |
| count_breakdown_cattle_patch | total cattle in breakdown herds per-patch | 698 | 5405 (2881) | 15,650 | 0 | 2955 (2130) | 12,029 | 487 | 2570 (1363) | 9,786 |
| count_milklicence_patch | total number of milk licences per-patch | 1 | 13 (8.8) | 43 | 0 | 13 (9) | 43 | 0 | 0 | 0 |
| count_reactors_patch | total number of reactors per-patch | 28 | 466 (368) | 2,016 | 0 | 214 (208) | 942 | 13 | 261 (225) | 1,193 |
| n_common_MLVA_type_patch | N. reactors with the most common MLVA type | 4 | 100 (91) | 449 | 0 | 87 (103) | 511 | 1 | 86 (89) | 514 |
| mean_count_breakdown_herd | mean breakdowns per-herd per-patch | 1 | 1 (0.2) | 2 | 0 | 1 (0.3) | 3 | 1 | 1 (0.15) | 2 |
| sum_breakdown_patch | total breakdowns per-patch | 11 | 61 (38) | 223 | 0 | 20 (15) | 76 | 6 | 42 (28) | 170 |
| mean_herd_size_patch | mean herd size per-patch | 35 | 132 (49) | 290 | 0 | 230 (82) | 573 | 35 | 93 (36) | 253 |
| mean_inwards_movement_herd | mean herd-level inwards movement intensity 6 months before breakdown | 0.04 | 0.15 (0.06) | 0.3 | 0 | 0.05 (0.04) | 0.27 | 0.05 | 0.19 (0.07) | 0.38 |
| sum_inwards_movement_patch | patch-level inwards movement intensity 6 months before breakdown | 0.03 | 0.26 (0.13) | 0.68 | 0 | 0.06 (0.06) | 0.43 | 0.02 | 0.37 (0.15) | 0.7 |
| mean_breakdown_length_herd | mean breakdown length per-herd per-patch (days) | 154 | 222 (35) | 350 | 0 | 227 (47) | 386 | 144 | 222 (43) | 477 |
| sum_breakdown_length_patch | total breakdown length per-patch (days) | 1891 | 13,705 (8811) | 52,824 | 0 | 4594 (3789) | 18,180 | 866 | 9298 (6310) | 39,109 |
| y | patch centroid (/100) | 3221 |  | 4516 | 3166 |  | 4441 | 3221 |  | 4516 |
| x | patch centroid(/100) | 2100 |  | 3646 | 2190 |  | 3635 | 2100 |  | 3646 |

**Table S3**: AIC values for each of the models, compared to null models containing only the “total reactors” variable.

| Model | AIC Final Model | AIC Null Model |
| --- | --- | --- |
| breakdown level (all data) | 17,117 | 17,452 |
| breakdown level (dairy only) | 5,433 | 5,460 |
| breakdown level (non-dairy only) | 11,682 | 11,961 |
| herd level (all data) | 14,095 | 15,610 |
| herd level (dairy only) | 4,175 | 4,296 |
| herd level (non-dairy only) | 9,916 | 10,684 |
| patch level (all data) | 660 | 686 |
| patch level (dairy only) | 566 | 602 |
| patch level (non-dairy only) | 643 | 698 |

**Table S4**: Final full model for the breakdown level analysis constructed using all data.

| **Variable** | **Estimate** | **Std. Error** | **z value** | **p** | **IRR** | **95%CI L** | **95%CI U** |
| --- | --- | --- | --- | --- | --- | --- | --- |
| Intercept | 0.204 | 0.011 | 19.179 | <0.001 | 1.226 | 1.200 | 1.251 |
| total_reactors_over_breakdown | 0.069 | 0.008 | 9.222 | <0.001 | 1.072 | 1.056 | 1.088 |
| herd_size | 0.078 | 0.010 | 7.874 | <0.001 | 1.081 | 1.060 | 1.102 |
| inwards_movement_intensity | 0.091 | 0.010 | 9.013 | <0.001 | 1.095 | 1.073 | 1.116 |
| breakdown_length_days | 0.098 | 0.008 | 11.823 | <0.001 | 1.103 | 1.085 | 1.121 |
| y_latitude | -0.022 | 0.011 | -2.081 | 0.038 | 0.978 | 0.958 | 0.991 |
| herd_size:inwards_movement_intensity | 0.058 | 0.010 | 5.976 | <0.001 | 1.059 | 1.039 | 1.080 |

**Table S5**: Final full model for the breakdown level analysis constructed using only data from herds with milk licences.

| **Variable** | **Estimate** | **Std. Error** | **z value** | **p** | **IRR** | **95%CI L** | **95%CI U** |
| --- | --- | --- | --- | --- | --- | --- | --- |
| Intercept | 0.208 | 0.019 | 11.153 | <0.001 | 1.231 | 1.187 | 1.277 |
| total_reactors_over_breakdown | 0.094 | 0.014 | 6.723 | <0.001 | 1.099 | 1.068 | 1.128 |
| herd_size | 0.048 | 0.018 | 2.728 | 0.006 | 1.050 | 1.013 | 1.086 |
| breakdown_length_days | 0.078 | 0.016 | 4.872 | <0.001 | 1.081 | 1.047 | 1.115 |

**Table S6**: Final full model for the breakdown level analysis constructed using only data from herds without milk licences.

| **Variable** | **Estimate** | **Std. Error** | **z value** | **p** | **IRR** | **95%CI L** | **95%CI U** |
| --- | --- | --- | --- | --- | --- | --- | --- |
| Intercept | 0.185 | 0.013 | 14.424 | <0.001 | 1.204 | 1.174 | 1.234 |
| total_reactors_over_breakdown | 0.071 | 0.010 | 7.504 | <0.001 | 1.074 | 1.054 | 1.094 |
| herd_size | 0.061 | 0.012 | 5.283 | <0.001 | 1.063 | 1.039 | 1.088 |
| inwards_movement_intensity | 0.084 | 0.012 | 6.885 | <0.001 | 1.088 | 1.062 | 1.114 |
| breakdown_length_days | 0.095 | 0.010 | 9.054 | <0.001 | 1.100 | 1.077 | 1.122 |
| herd_size:inwards_movement_intensity | 0.052 | 0.010 | 4.922 | <0.001 | 1.053 | 1.031 | 1.074 |

**Table S7**: Final full model for the herd level analysis constructed using all data.

| **DVO (Random effect)** | **Variance** | **Std.Dev.** |  |  |  |  |  |
| --- | --- | --- | --- | --- | --- | --- | --- |
|  | 0.0013 | 0.037 |  |  |  |  |  |
|  |  |  |  |  |  |  |  |
| **Variable** | **Estimate** | **Std. Error** | **z value** | **p** | **IRR** | **95%CI L** | **95%CI U** |
| Intercept | 0.439 | 0.017 | 25.541 | <0.001 | 1.552 | 1.500 | 1.605 |
| total_reactors_herd | 0.091 | 0.014 | 6.403 | <0.001 | 1.095 | 1.065 | 1.125 |
| n_common_MLVA_type_herd | -0.042 | 0.015 | -2.900 | 0.004 | 0.959 | 0.932 | 0.992 |
| mean_herd_size | 0.155 | 0.010 | 15.152 | <0.001 | 1.167 | 1.144 | 1.191 |
| count_breakdown_herd | 0.175 | 0.009 | 18.577 | <0.001 | 1.192 | 1.170 | 1.214 |
| mean_inwards_movement | 0.172 | 0.010 | 17.475 | <0.001 | 1.188 | 1.165 | 1.211 |
| mean_breakdown_length | 0.094 | 0.009 | 10.174 | <0.001 | 1.098 | 1.079 | 1.118 |
| mean_herd_size:mean_inwards_movement | 0.093 | 0.009 | 10.858 | <0.001 | 1.098 | 1.079 | 1.116 |
| mean_herd_size: mean_breakdown_length | -0.017 | 0.005 | -3.317 | 0.001 | 0.984 | 0.974 | 0.993 |

**Table S8**: Final full model for the herd level analysis constructed using data from herds with milk licences.

|  |  |  |  |  |  |  |  |
| --- | --- | --- | --- | --- | --- | --- | --- |
| **Variable** | **Estimate** | **Std. Error** | **z value** | **p** | **IRR** | **95%CI L** | **95%CI U** |
| Intercept | 0.511 | 0.020 | 25.201 | <0.001 | 1.667 | 1.602 | 1.734 |
| n_common_MLVA_type_herd | -0.064 | 0.032 | -2.009 | 0.040 | 0.938 | 0.882 | 0.981 |
| mean_herd_size | 0.124 | 0.018 | 6.842 | <0.001 | 1.131 | 1.092 | 1.172 |
| count_breakdown_herd | 0.167 | 0.018 | 9.230 | <0.001 | 1.182 | 1.140 | 1.224 |
| mean_breakdown_length | 0.061 | 0.019 | 3.248 | 0.001 | 1.063 | 1.024 | 1.102 |
| mean_inwards_movement | 0.070 | 0.019 | 3.648 | <0.001 | 1.073 | 1.033 | 1.114 |
| mean_herd_size: mean_inwards_movement | 0.043 | 0.019 | 2.273 | 0.023 | 1.044 | 1.006 | 1.084 |

**Table S9**: Final full model for the herd level analysis constructed using data from herds without milk licences.

| **DVO (Random effect)** | **Variance** | **Std.Dev.** |  |  |  |  |  |
| --- | --- | --- | --- | --- | --- | --- | --- |
|  | 0.0011 | 0.033 |  |  |  |  |  |
|  |  |  |  |  |  |  |  |
|  | **Estimate** | **Std. Error** | **z value** | **p** | **IRR** | **95%CI L** | **95%CI U** |
| Intercept | 0.394 | 0.018 | 21.503 | <0.001 | 1.484 | 1.431 | 1.538 |
| total_reactors_herd | 0.075 | 0.009 | 8.178 | <0.001 | 1.078 | 1.059 | 1.098 |
| mean_herd_size | 0.125 | 0.013 | 9.821 | <0.001 | 1.134 | 1.106 | 1.162 |
| count_breakdown_herd | 0.170 | 0.011 | 15.361 | <0.001 | 1.186 | 1.160 | 1.212 |
| mean_inwards_movement | 0.165 | 0.012 | 13.678 | <0.001 | 1.179 | 1.152 | 1.207 |
| mean_breakdown_length | 0.080 | 0.010 | 8.113 | <0.001 | 1.083 | 1.063 | 1.105 |
| mean_herd_size:mean_inwards_movement | 0.067 | 0.009 | 7.874 | <0.001 | 1.069 | 1.052 | 1.087 |
| mean_herd_size:mean_breakdown_length | -0.024 | 0.006 | -3.933 | <0.001 | 0.976 | 0.965 | 0.988 |

**Table S10**: Final full model for the patch level analysis constructed using data from all herds.

|  | **Estimate** | **Std. Error** | **z value** | **p** | **IRR** | **95%CI L** | **95%CI U** |
| --- | --- | --- | --- | --- | --- | --- | --- |
| Intercept | 2.712 | 0.024 | 114.959 | <0.001 | 15.050 | 14.370 | 15.770 |
| sum_total_reactors_in_patch | 0.208 | 0.020 | 10.481 | <0.001 | 1.230 | 1.180 | 1.280 |
| inwards_movement_intensity_patch | 0.079 | 0.024 | 3.322 | 0.001 | 1.080 | 1.030 | 1.130 |
| mean_herd_MLVA_richness | 0.070 | 0.027 | 2.641 | 0.008 | 1.070 | 1.020 | 1.130 |

**Table S11**: Final full model for the patch level analysis constructed using only data from herds with milk licences.

|  | **Estimate** | **Std. Error** | **z value** | **p** | **IRR** | **95%CI L** | **95%CI U** |
| --- | --- | --- | --- | --- | --- | --- | --- |
| Intercept | 2.029 | 0.038 | 53.946 | <0.001 | 7.610 | 7.070 | 8.190 |
| sum_total_reactors_in_patch | 0.101 | 0.042 | 2.426 | 0.015 | 1.110 | 1.020 | 1.200 |
| count_breakdown_cattle_patch | 0.219 | 0.041 | 5.391 | <0.001 | 1.240 | 1.150 | 1.350 |
| mean_herd_MLVA_richness | 0.132 | 0.034 | 3.917 | <0.001 | 1.140 | 1.070 | 1.220 |

**Table S12**: Final full model for the patch level analysis constructed using only data from herds without milk licences.

|  | **Estimate** | **Std. Error** | **z value** | **p** | **IRR** | **95%CI L** | **95%CI U** |
| --- | --- | --- | --- | --- | --- | --- | --- |
| Intercept | 2.607 | 0.031 | 85.057 | < 0.001 | 13.560 | 12.770 | 14.400 |
| sum_total_reactors_in_patch | 0.213 | 0.022 | 9.827 | < 0.001 | 1.240 | 1.190 | 1.290 |
| inwards_movement_intensity_patch | 0.076 | 0.025 | 3.086 | 0.002 | 1.080 | 1.030 | 1.130 |
| mean_herd_MLVA_richness | 0.135 | 0.027 | 4.961 | <0.001 | 1.140 | 1.090 | 1.210 |
